# Endoscopic submucosal dissection for a large (exceeding 3 cm) exophytic gastric schwannoma with 24-hour intragastric retention: a case report and brief literature review

**DOI:** 10.3389/fgstr.2025.1703551

**Published:** 2026-07-31

**Authors:** Rong Xie, Weiwei Zhang, YanningI Mao, Yi Qin, Jiaxu Wang, Qi Luo, Linhua Teng, Jiawen Liu

**Affiliations:** Department of Gastroenterology, The First People’s Hospital of Nanning, Nanning, China

**Keywords:** endoscopic submucosal dissection, gastric schwannoma, exceeding 3 cm, exophytic, S100

## Abstract

**Background:**

The clinical role of endoscopic resection in the treatment of exophytic gastric schwannomas measuring ≥3 cm remains controversial owing to technical difficulties and a paucity of evidence.

**Case presentation:**

A 58-year-old female patient was admitted for treatment after a gastric mass was discovered during routine physical examination. Endoscopic ultrasonography (EUS) revealed a well-demarcated, hypoechoic submucosal tumor measuring 40.9×32.3 mm. The patient subsequently underwent endoscopic submucosal dissection (ESD) under general anesthesia. Histopathological analysis confirmed the diagnosis of gastric schwannoma, with immunohistochemical staining showing strong S-100 protein positivity. The patient had an uneventful recovery with no procedure-related complications, and there has been no evidence of recurrence to date.

**Conclusion:**

While this single-case experience indicates the technical feasibility of ESD for gastric schwannomas >3 cm, larger-scale prospective studies are required to establish its role in clinical practice.

## Introduction

Gastric schwannomas (GS), originating from the enteric nerve plexus, represent a rare but increasingly recognized subtype of gastric mesenchymal tumors, with reported incidence likely rising due to widespread use of diagnostic upper endoscopy. Epidemiological studies demonstrate a slight female (1) predominance (male-to-female ratio 9:7) (2, 3)and mean diagnosis age of 50.4 years, with 62.5% of cases being asymptomatic incidental findings. Diagnostic challenges include the limited sensitivity of endoscopic biopsies (frequently yielding false-negative results (4)) and nonspecific imaging features on CT/MRI, typically showing exophytic or mixed-growth masses without hemorrhage, necrosis or degenerative changes. Characteristic EUS findings of well-circumscribed, heterogeneous hypoechoic/isoechoic submucosal lesions lacking cystic changes or calcifications provide important diagnostic clues (5–7), though definitive differentiation from GISTs often requires postoperative pathology. Immunohistochemistry remains the diagnostic gold standard (2, 3, 8), with GS demonstrating strong diffuse S100 positivity while being negative for CD34, CD117, DOG-1, calretinin, SMA and desmin - a profile that reliably distinguishes GS from other gastric stromal tumors. The clinical symptoms of GS are nonspecific, and there are no obvious clinical signs. Unlike other tumors, GS usually does not exhibit hemorrhage, necrosis, cystic changes, or calcification (9–11). Due to these overlapping features, it is often challenging to differentiate GS from gastrointestinal stromal tumors (GISTs) based solely on imaging findings (12). Therefore, S100 immunoreactivity is the gold standard for the definitive diagnosis of this disease (13).

GS are benign neoplasms where surgical resection remains the primary treatment modality (14). However, considerable controversy persists regarding optimal surgical strategies (endoscopic resection, local excision, or extended surgery), particularly for larger tumors (diameter >3 cm) or those with extraluminal growth patterns (15). Previous studies suggest that larger GSs may present technical challenges during resection due to their rich vascular supply or dense adhesions to surrounding tissues, raising concerns about the feasibility and safety of endoscopic approaches. Furthermore, the standardized protocol for specimen retrieval following endoscopic resection (e.g., 24-hour gastric digestion prior to extraction) and its potential impact on pathological evaluation remains poorly characterized in current literature.

## Case presentation

**Figure 1:**
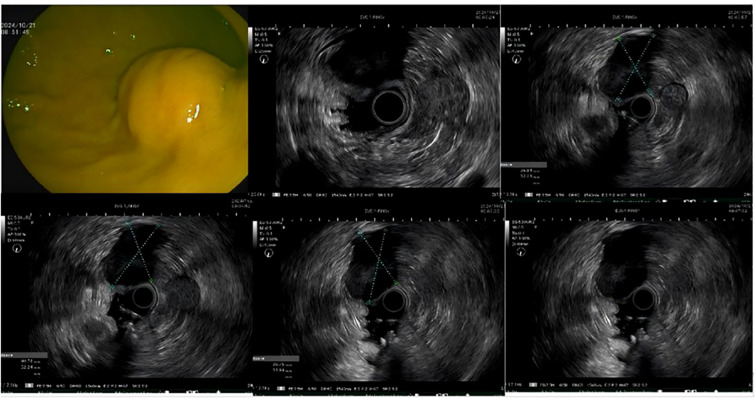
Ultrasound gastroscopy image.

**Figure 2:**
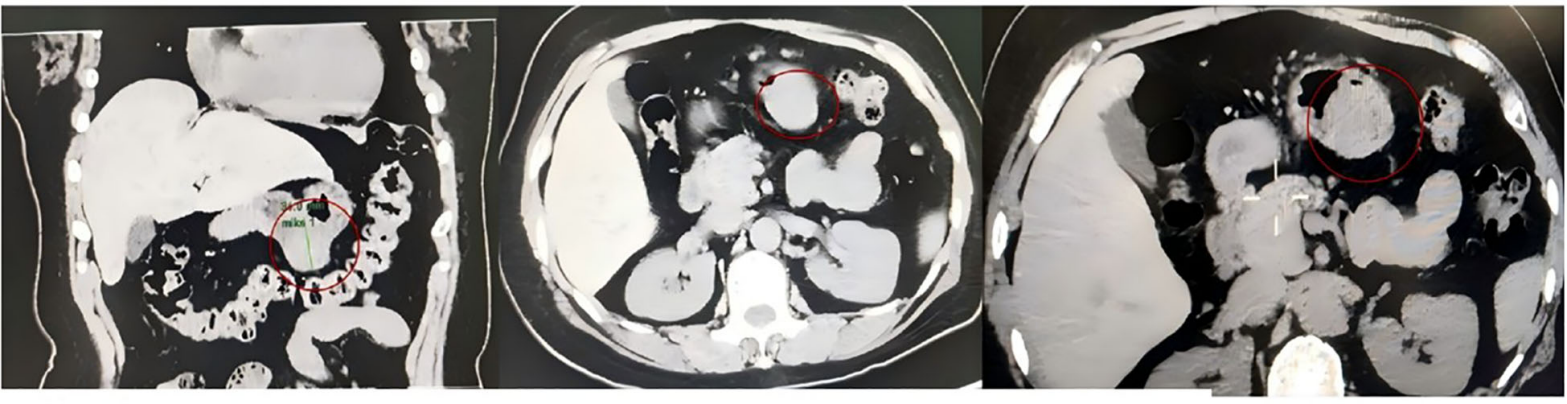
CT image.

**Figure 3:**
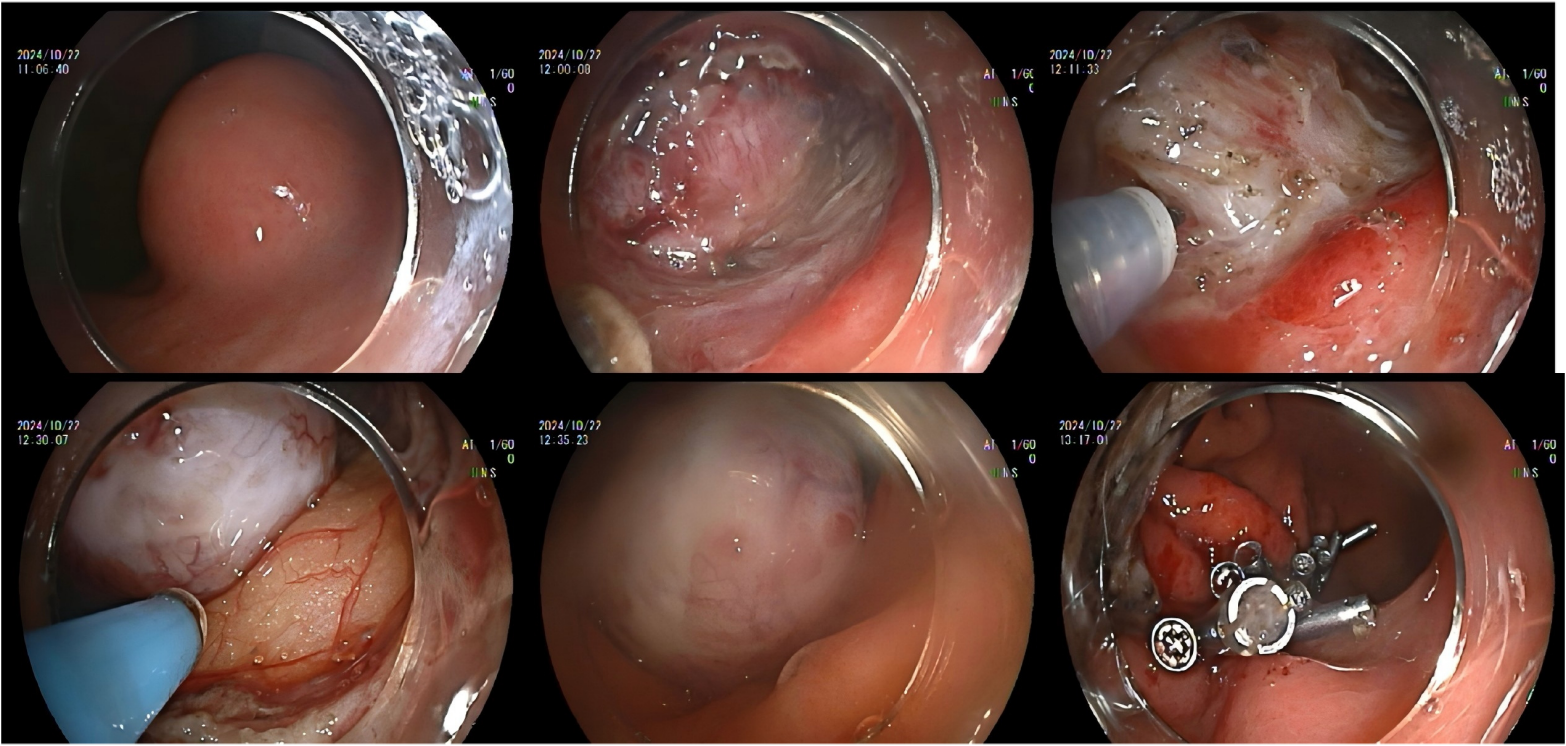
Surgery picture.

**Figure 4:**
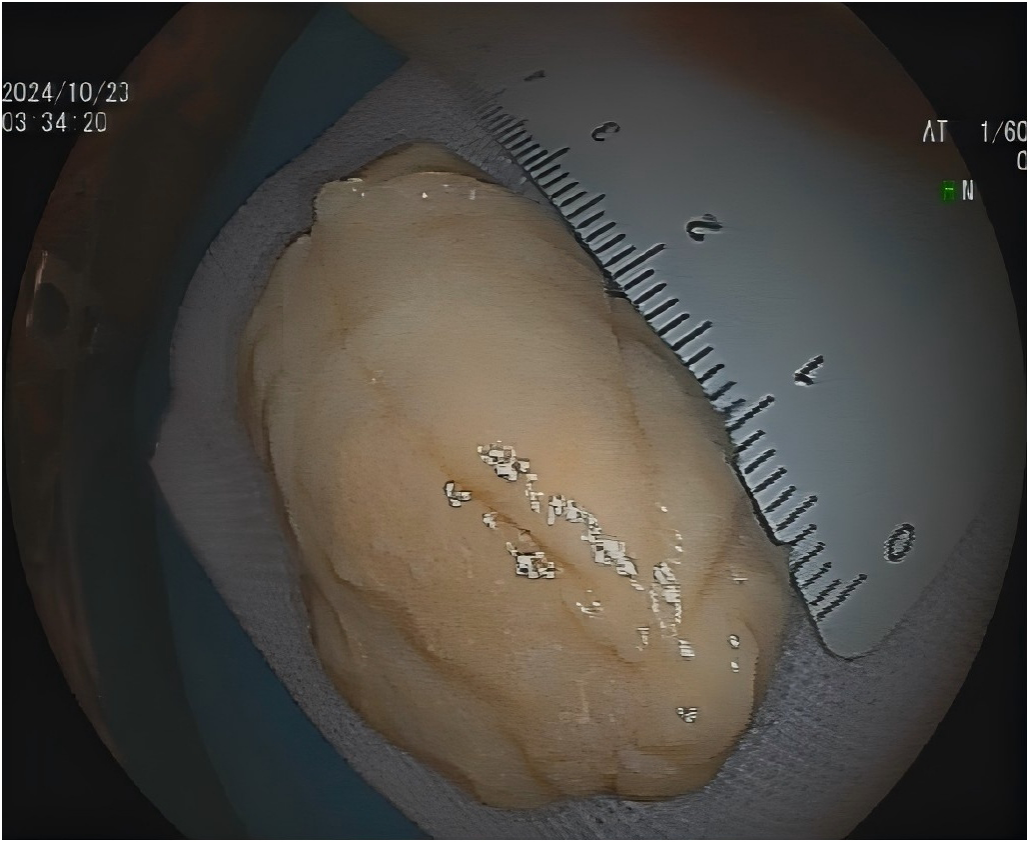
The tumor was softened and removed under endoscopy after 24 hours.

**Figure 5:**
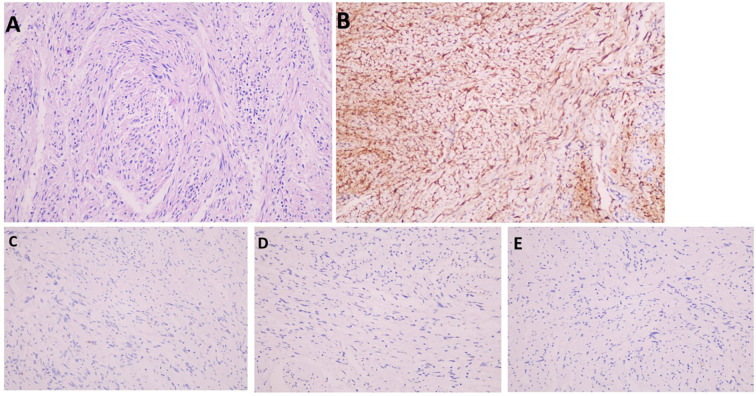
Pathological and immunohistochemical (IHC) images. **(A)** Numerous spindle-shaped cells are observed, ×20. **(B)** Shows S-100 strong positive, ×20. **(C)** Shows CD117 negative, ×20. **(D)** Shows DOG-1 negative, ×20. **(E)** Shows H-Caldesmon negative, ×20.

Endoscopic ultrasonography (EUS) identified a 4-cm exophytic mass originating from the muscularis propria layer, suggestive of a gastrointestinal stromal tumor (GIST, **Figure 1**). Preoperative CT (**Figure 2**) examination revealed a soft tissue density lesion within the gastric wall of the body, demonstrating well-defined margins and homogeneous attenuation with an unenhanced CT value of approximately 37 HU. The lesion measured about 3.0 cm × 3.5 cm × 3.4 cm and exhibited mixed intraluminal and extraluminal growth patterns. These imaging features are suggestive of a gastric space-occupying lesion, most likely representing a neoplastic process such as a gastrointestinal stromal tumor (GIST). Preoperative evaluations, including hepatic/renal function, coagulation profile, electrolytes, electrocardiography, and cardiac ultrasonography, were unremarkable. Tumor markers were all negative. The patient underwent endoscopic submucosal dissection (ESD) under general anesthesia with endotracheal intubation and CO_2_ insufflation. Gastroscopic examination showed a smooth-surfaced mucosal protrusion in the gastric body, with EUS findings consistent with a suspected GIST. Magnifying endoscopy with blue laser imaging (BLI) and indigo carmine staining revealed no abnormalities in glandular architecture (MS-, M-). After circumferential marking with a Dual knife, submucosal injection of indigo carmine and sodium hyaluronate achieved adequate lifting. A circumferential incision was made using a mucosal incision knife, exposing a whitish submucosal tumor originating from the serosal layer. The lesion was completely enucleated using an IT knife via submucosal dissection. During the procedure, perforation and bleeding occurred and were successfully managed with hot biopsy forceps for hemostasis. The tumor, measuring approximately 4.0 cm in diameter with a firm consistency and round morphology, was resected via full-thickness dissection using a snare. Hemostasis was achieved using hot biopsy forceps and hemostatic powder spray. The surgical field was then closed with multiple hemostatic clips. The entire procedure lasted 120 minutes with an estimated blood loss of 50 ml (**Figure 3**, **Supplementary Video 1**). The resected specimen remained in the gastric lumen and was spontaneously expelled after 24 hours due to softening (**Figure 4**). Histopathological examination revealed a 3.5 cm tumor composed of spindle cells arranged in fascicular and whorled patterns. The cells exhibited abundant eosinophilic cytoplasm with oval to spindle-shaped nuclei. Mitotic activity was low (<2/10 HPF). The stroma showed prominent thick-walled blood vessels and focal hyaline degeneration without necrosis. Immunohistochemical staining was positive for S-100 and SDHB, while negative for CK, LCA, CD117, CD34, DOG1, and H-Caldesmon. The Ki-67 proliferation index was approximately 1% (**Figures 5A–E**). Special stains (VG, PAS, methylene blue, carmine) were negative. These findings confirmed the diagnosis of gastric schwannoma. Postoperative management includes fasting, acid suppression, gastric mucosa protection, fluid replacement, 24-hour antibiotic administration for infection prevention, and vital signs monitoring. The patient resumed oral intake after 72 hours and was discharged on postoperative day 5 without complications such as hemorrhage, perforation, or mortality. The patient has now been followed up for 13 months with no signs of tumor recurrence, and continued surveillance is ongoing.

## Discussion

Extensive prior studies have conclusively established the high safety profile and favorable short- and long-term outcomes of endoscopic resection for gastric schwannomas (GS) smaller than 3 cm (15) (**Table 1**). These procedures are consistently associated with high R0 resection rates, manageable complications, and no reported recurrence or metastasis during mid- to long-term follow-up, thereby solidifying the evidence base for minimally invasive management of these tumors and establishing the <3 cm size criterion as a widely accepted safety threshold (16, 17). In contrast, evidence regarding the safety and feasibility of endoscopic resection for lesions exceeding this size threshold (e.g., 3.5 cm) remains scarce and inconclusive. This study presents the first systematic report of a successfully and completely resected 3.5 cm GS via endoscopy. Our experience demonstrates that, in high-volume centers equipped with advanced expertise, a meticulous preoperative workup—particularly endoscopic ultrasound (EUS) confirming a muscularis propria origin without features suggestive of malignancy—coupled with proactive endoscopic closure of the muscle layer defect, can make endoscopic resection a safe and effective therapeutic alternative for selected lesions marginally larger than 3 cm. These findings suggest the potential for cautiously expanding the indications for endoscopic resection to include larger tumors in the future. In this study, we present a 3.5 cm, predominantly exophytic gastric schwannoma successfully resected via endoscopic submucosal dissection (ESD). The defect was closed with titanium clips, and the specimen was retained in the gastric lumen for 24 hours before fragmented retrieval—a deliberate delay to mitigate perforation risk. This approach avoided intraoperative tumor displacement, perforation, or infection, demonstrating that delayed extraction may safely overcome traditional size limitations. Our findings propose a feasible minimally invasive strategy for submucosal gastric tumors >3 cm, expanding the scope of endoscopic management.

**Table 1:** Summary of reported cases of endoscopic resection for gastric schwannoma > 3 cm.

Author (year)	Tumor size (cm)	Location	Resection method	En bloc resection (R0)	Complications	Follow-up (years)	Recurrence
Carla Saoud (2022) (13)	2.2~8.6 cm	gastric	–	Yes	NO	–	NO
Zhihan Zhong (2022) (1)	2~5 cm	gastric	lapa-roscopic gastrectomy+ESD+robotic gastrectomy	Yes	NO	1~9	NO
F. S. Meng (2015)	0.6~6.5 cm	gastric	esd	Yes	perforation	0.5-2	NO
Ming-Yan Cai (2015) (14)	0.3~4.0 cm	gastric	ESD+EFTR	85.7%	perforation	0.25-0.5	NO

Endoscopy has been widely accepted as a viable alternative for the resection of gastric submucosal tumors (SMTs), offering both diagnostic accuracy and complete excision. Previous studies have demonstrated the safety and efficacy of endoscopic treatment for gastric schwannomas (GS) (17–19). Li (17) et al. reported five cases of GS successfully resected endoscopically, with all tumors completely removed (mean procedure time: 57.6 min). During a mean follow-up of 7.4 months, no recurrence was observed. Similarly, Hu (19) et al. achieved R0 resection in all 14 GS cases without complications. In a retrospective study by Cai (18) et al. involving 14 patients, eight underwent full-thickness resection, while six underwent submucosal dissection, yielding an overall complete resection rate of 85.7% (12/14). One case required partial gastectomy due to tumor location (gastric angle) and predominant extraluminal growth. In the present case, the tumor exhibited mostly extraluminal growth. Following endoscopic full-thickness resection and clip closure of the defect, the patient recovered well without gas-related complications. No delayed bleeding, perforation, or postprandial symptoms (e.g., pain or dysphagia) were observed during follow-up, aligning with the safety profiles reported in prior studies.

According to authoritative textbooks of surgical pathology (e.g., Rosai and Ackerman’**s S**urgical Pathology), fibrous-rich mesenchymal tissues, such as fibromatosis and schwannomas, can maintain their histological architecture for a longer period ex vivo compared to epithelial or parenchymal organ tissues due to their abundant extracellular matrix and relatively low cellular density. Typically, they retain sufficient diagnostic value for 24 to 48 hours. This case demonstrates the feasibility and safety of endoscopic submucosal dissection (ESD) combined with 24-hour intragastric specimen retention for en bloc resection of a large (>3 cm) predominantly extraluminal gastric schwannoma, achieving complete tumor removal without laparoscopic assistance. Moreover, postoperative pathological sections clearly revealed spindle-shaped cells, and immunohistochemical staining demonstrated strongly positive for the specific diagnostic marker S-100. These findings confirm that the 24-hour intragastric retention did not compromise the subsequent diagnosis of the gastric schwannoma.

## Data Availability

The original contributions presented in the study are included in the article/**Supplementary Material**. Further inquiries can be directed to the corresponding author.

## Supplementary material

Supplementary Video 1 Video clip of endoscopic surgery for resection of a tumor growing outside the gastric cavity.
